# Physiological and Molecular Responses of *Pyrus pyraster* Seedlings to Salt Treatment Analyzed by miRNA and Cytochrome P450 Gene-Based Markers

**DOI:** 10.3390/plants13020261

**Published:** 2024-01-16

**Authors:** Viera Paganová, Marek Hus, Helena Lichtnerová, Jana Žiarovská, Dagmar Moravčíková, Matúš Kučka, Katarína Ražná, Aqsa Abbas

**Affiliations:** 1 Institute of Landscape Architecture, Faculty of Horticulture and Landscape Engineering, Slovak University of Agriculture, 949 76 Nitra, Slovakia; marek.hus@uniag.sk (M.H.); helena.lichtnerova@uniag.sk (H.L.); 2 Institute of Plant and Environmental Sciences, Faculty of Agrobiology and Food Resources, Slovak University of Agriculture, 949 76 Nitra, Slovakia; jana.ziarovska@uniag.sk (J.Ž.); xmoravcikova@uniag.sk (D.M.); xkucka@uniag.sk (M.K.); katarina.razna@uniag.sk (K.R.); xabbas@uniag.sk (A.A.)

**Keywords:** salinity, woody plant, adaptability, DNA polymorphism, miRNA-based markers, PBA technique

## Abstract

Physiological and molecular marker-based changes were studied in the tissues of two-year-old *Pyrus pyraster* (L.) Burgsd. seedlings under salt treatment. For 60 days, 5 mL of 100 mM NaCl solution was applied to each plant per day to a cumulative volume of 300 mL in the substrate. In response to osmotic stress, the seedlings increased their water use efficiency (WUE) on day 20 of regular NaCl application and maintained a stable net photosynthetic rate (A_n_) per unit area. Under conditions of increasing salinity, the young plants maintained a balanced water regime of the leaf tissues (Ψ_wl_). The seedlings invested mass to their root growth (R/S), retained a substantial portion (72%) of Na^+^ ions in the roots, and protected their leaves against intoxication and damage. A significant decrease in the leaf gas exchange parameters (g_s_, E, A_n_) was manifested on day 60 of the experiment when the cumulative NaCl intake was 300 mL per plant. The variability in the reactions of the seedlings to salinity is related to the use of open-pollinated progeny (54 genotypes) in the experiment. Lus-miR168 showed tissue- and genotype-specific genome responses to the applied stress. Polymorphic miRNA-based loci were mostly detected in the root samples on the 20th and 35th days of the experiment. The cumulative effect of the salt treatment was reflected in the predominance of polymorphic loci in the leaves. We can confirm that miRNA-based markers represent a sensitive detection tool for plant stress response on an individual level. The screening and selection of the optimal type of miRNA for this type of research is crucial. The cytochrome P450-Based Analog (PBA) techniques were unable to detect polymorphism among the control and treated seedlings, except for the primer pair CYP2BF+R, where, in the roots of the stressed plant, insertions in the amplicons were obtained. The expression ratios of cytochrome P450 in the salt-stressed plants were higher in the roots in the case of 20/100 mL and in the leaves with higher doses. The observed physiological and molecular responses to salinity reflect the potential of *P. pyraster* seedlings in adaptation to osmotic and ionic stress.

## 1. Introduction

Wild pear *Pyrus pyraster* (L.) Burgsd. grows in different types of habitats, including woodlands, scattered vegetation, meadows, and pasturelands in open landscapes over a temperate zone in Europe [1,2]. This tree has a relatively wide ecological amplitude [3]. It can be found in a wide variety of soil types and various subsoils, mainly basic rocks [4,5,6]. *P. pyraster* is a light-demanding species that often grows in rather extreme site conditions where competition with other tree species is weakened [1,6,7]. Recent studies have confirmed the high phenotypic variability of the European wild pear [8,9] and emphasized phenotypic plasticity as a major driver in the adaptation of *P. pyraster* to rapid climate change [10].

There are little data in the literature on the responses of *P. pyraster* to environmental stressors. Our previous experimental research focused on the responses of *P. pyraster* seedlings to drought [11] and salinity [12,13]. The impact of different salt concentrations on the growth of *P. pyraster* seedlings, the content of assimilation pigments, and the sodium ion distribution were studied in the juvenile stage of growth. Significant reductions in the dry masses of the leaves, stems, and roots were documented in the treatment with a higher concentration of NaCl (120 mM). Under a low-salinity treatment (60 mM NaCl), *P. pyraster* invested more dry matter in the roots and maintained a balanced total dry weight per plant [12]. Further study documented the significant role of the root system in the resistance of woody plants and in ensuring their survival in conditions of excessive salinity [13]. The investment in root growth improved the water supply of *P. pyraster* seedlings, enhanced the retention of salt ions in the root system, and restricted their transfer and accumulation in leaves. This study showed that *P. pyraster* can cope with salinity, but deeper study on a molecular level is necessary for a better understanding of the mechanisms of salinity tolerance. Genomic polymorphism underlies the adaptive potential of plants to different climatic conditions. Understanding and mapping plant genome variation using molecular markers is a prerequisite for broadening the genetic base of crops and reducing their sensitivity to adverse environmental conditions [14] and among different types of analysis, genetic polymorphism was involved as an effective way to analyze the response of plants to stress salinity.

Whole-genome polymorphism profiles of plants are still relevant in broad application areas, from the basic characteristics of varieties to specific issues of association mapping [15,16]. In this study, DNA-based markers that analyze the length polymorphism among cytochrome P450 sequences were used for the whole-genome mapping of pear genotypes growing under salt stress. Cytochrome P450 genes are abundantly represented in the plant genome, which provides a background for the development of polymorphic genetic markers that are based on universally applicable primers for the cytochrome P450 mono-oxygenase analog, which is designed to flank intron regions [17]. In plants, mono-oxygenases participate in the processes of oxidative detoxification and the biosynthesis of secondary metabolites, and they have also been described as part of the stress-response modulating mechanisms, making them suitable stress-sensitive genomic markers [18]. The cytochrome P450 (CYP) superfamily is a part of the basic processes promoting plant growth and development and protecting plants from stresses via multiple biosynthetic and detoxification pathways [18,19,20].

Other types of markers that were used in this study were microRNA markers. MiRNAs are endogenous 19–24 bp long stretches of non-coding single-stranded RNA that regulate gene expression by inhibiting gene translation or promoting cleavage of the target post-transcriptional mRNA. They play an important role in plant responses to abiotic stresses of various natures, including low temperature, drought, salinity, oxidative stress, UV-B radiation, heavy metals, etc. [21,22]. They are particularly important in plant growth and development, hormone regulation, organ differentiation, alternative assembly, and metabolite accumulation [23,24]. Their activity also depends on the concentration of nutrients in the soil or nutrient solution [25]. Functional markers based on microRNA molecules represent a highly efficient, stable, reproducible, low-cost, and protocol-transferable procedure for genomic polymorphism mapping [26,27]. Polymorphisms detected by the application of miRNA markers indicate changes in the sequences of miRNA loci, due to changes in the regulation of target genes [28]. A genome-wide identification of *Pyrus pyrifolia* (Nakai) revealed 186 conserved pear miRNAs belonging to 37 families. Potential target genes included transcription factors and stress-responsive genes [29]. In *Pyrus pyrifolia* (Nakai), miR156 controlling an age-dependent flowering pathway was studied [30], and in *Pyrus sinkiangensis* (Yu), novel miRNAs in the flower organs were identified [31]. MicroRNA profiles of pear fruit (*Pyrus bretschneideri*) were investigated during different developmental stages to identify miRNAs involved in pear fruit development and quality [32]. Regarding biotic stress response, high-temperature-altered miRNAs were identified from *Pyrus pyrifolia* from shoots grown in vitro and infected with apple stem grooving virus (ASGV). These miRNAs mediated the regulation of the target genes in meristematic cells [33]. Long-noncoding RNAs (lncRNAs) are precursors of miRNAs. Genome-wide analysis of lncRNAs revealed 261 pear miRNAs. The results suggest that the lncRNA–miRNA–mRNA regulatory network plays an important role in flower bud dormancy regulation during cold stress [34] 

The aim of this study was to analyze the effect of salt stress conditions on the tissues of young seedlings of *P. pyraster* based on physiological and molecular markers. We were interested in the young seedlings’ response to abiotic stress to test their ability to adjust to their natural surrounding environment and to improve the pears’ resistance to salinity conditions. Molecular markers enable us to detect the stress symptoms at earlier stages and evaluate the salinity impact on pear seedlings. 

## 2. Material and Methods

### 2.1. Plant Material

The experimental plants represent open-pollinated progenies suitable for planting in shelterbelts, as well as for forestry purposes. The two-year-old seedlings were the offspring of a mother tree growing in an open landscape at an altitude of 570 m (Tŕnie, Slovakia, latitude 48.604490, longitude 19.022024). The site conditions of the submontane altitudinal zone represent the ecological optimum for this taxon. According to the classification of the climatic regions of Slovakia [35], this locality belongs to a moderately warm region with 750 mm of precipitation, an average January temperature of −3.0 °C, and an average July temperature of 18 °C. The seedlings were placed in plastic pots (volume of 1 L) containing a fertilized peat-based growth substrate (20% black peat and 80% white peat moss, 0–5 mm fraction, pH 5.5–6.5, enriched with nutrients at 1.0 kg/m^3^ NPK 14:16:18). The pots were covered with a plastic bag to avoid uncontrolled leakage of the saline solution and the irrigation dose.

### 2.2. Experimental Design

In the experiment, 54 seedlings (genotypes) of *P. pyraster* were studied; half of them underwent salt treatment, and the rest were control plants. The water content of the substrate was calculated based on the wet weight [36] and maintained at 80% of the weight of the fully saturated substrate.

$$Mn=\frac{\left(Ww-Wd\right)}{Ww}\times 100$$

*Mn* = moisture content (%) of the material *n*;
*Ww* = wet weight of the sample;
*Wd* = weight of the sample after drying.

The water regime was maintained and regulated on a gravimetric principle by regular weighing of the containers on a precision industrial scale (Kern & Sohn GmbH, Balingen, Germany) with laboratory accuracy (max = 8000 g, standard deviation = 0.05 g) at 2-day intervals. The salinity treatment was applied by adding 5 mL of NaCl solution (100 mM NaCl with 10.1 dS·m^−1^ of electric conductivity) per plant per day. The total amount of saline solution applied in the experiment was 300 mL per plant. The control plants were saturated with water. The experiment was conducted in the PolEko KK1450 growth chamber (POL-EKO-APARATURA sp.j., Wodzisław S´ląski, Poland) under a regulated environment with a 14/10 h photoperiod, 250 μmol m^−2^·s^−1^ of irradiation density, and 65% air humidity. During the light period, the temperature was maintained at 24 °C; during the dark period, the temperature was maintained at 14 °C. After 20 days of acclimatization, the plants were treated daily with a NaCl solution for 60 days from June to July.

The measurements of the morphometric traits and the distribution of the fresh and dry mass in the plant organs were performed at the beginning and end of the experiment. The leaf gas exchange parameters (g_s_, A_n_, E, WUE), the water potential of the leaf tissue (Ψ_wl_), and the relative water content (RWC) were measured on the 1st, 20th, 35th, 50th, and 60th day of the experiment after total applied doses of 0, 100, 175, 250, and 300 mL of NaCl solution, respectively.

### 2.3. Measurement and Analysis of Plant Parameters

The assessment of the plant parameters was performed on 24 plants (12 salt-treated and 12 control). The total fresh mass of all individuals was determined before the seedlings were planted in the pots and at the end of the experiment. Before their weighing, the plant roots were gently extracted from the growth substrate by hand and carefully washed to minimize fine root loss.

The WinRhizo REG 2009 system (Regent Instruments, Québec, QC, Canada, SK0410192) was used for the measurement of the root length (mm). The length of the primary stem of the experimental plants was also measured, and the total leaf area (LA) was determined by scanning the fresh leaves using ImageJ software. The dry weight of the plant organs was determined after the plant material was dried at 105 °C until it reached a constant weight. Other parameters calculated were the leaf water content (LWC), the specific root length (SRL) [37], the specific leaf area (SLA), and the root-to-shoot ratio (R:S). The SLA was calculated as the ratio of the leaf area to the leaf dry mass [38].

### 2.4. Leaf Gas Exchange

The net photosynthetic rate (A_n_), stomatal conductance (g_s_), transpiration rate (E), and water use efficiency (WUE) were measured in the samples (n = 7) of the control and salt-treated seedlings at the beginning of the experiment, and then 20 days after the first application of 100 mM NaCl. The measurements were performed using the CIRAS-3 gasometer (PP-Systems, Amesbury, MA, USA) attached to a PLC3 universal leaf cuvette fitted with a 1.75 cm^2^ measurement window on the fully expanded leaf for each plant on the upper part of the seedling according to the protocol by Paganová et al. [13], modified based on protocols by Parsons et al. [39] and Hunt et al. [40].

### 2.5. Leaf Water Potential and Relative Water Content

The water potential of the leaf tissues (Ψ_wl,_) was determined by psychrometric measurement performed by Wescor (model Psypro, EliTech Inc., Logan, UT, USA) using a C-52 sample chamber at an ambient temperature of 21 °C from 7 a.m. to 3 p.m. The leaf samples were taken from 3 plants in 3 repetitions for each of the variants.

The relative water content (RWC; %) was determined by the gravimetric method according to Barrs and Weatherley [41], with 4 h of saturation of the leaf samples in water at 4 °C in the dark. The leaf samples were taken from 3 plants in 3 repetitions for each of the variants. The RWC was calculated as:

RWC = [(FW − DW)/(SW − DW)] × 100

FW = fresh weight;
DW = dry weight;
SW = weight after full saturation of the leaf samples.

### 2.6. Statistical Analysis

Mathematical and statistical data analyses were performed using the Statgraphics Centurion XVII software (StatPoint Technologies, Warrenton, VA, USA, XVIII, license number: B480-E10A-00EA-P00S-60PO). Analyses of the normality and homogeneity of variance for all variables were performed with Shapiro–Wilk’s test (at a significance level of α = 0.001) and Leven’s test (at a significance level of α = 0.05). Grubbs’ test was used to detect and remove single outliers in the experimental data set. The assessment of differences between the control plants and seedlings grown under salt treatment was performed by one-way ANOVA. The multiple comparison of means was performed using the Tukey honest significant difference (HSD) test (at significance levels of =0.05). Regression analysis was applied for the assessment of the relationships among the RWC, Ψ_wl_, and the cumulative salt uptake.

### 2.7. MicroRNA-Based Assay

The assay was conducted according to the protocol by Ražná et al. [42], modified based on the protocols by Fu et al. [26] and Yadav et al. [27]. In total, 15 genotypes were analyzed, including biological triplicates for each variant (control 1 (C1), control 2 (C2), stress application on the 20th (V1), 35th (V2), and 60th (V3) days). For each genotype, the leaf and root tissues were analyzed separately. The genomic DNA was isolated by applying the ISOLATE II Plant DNA Kit (Bioline Meridian Bioscience, London, UK), and the RNA was isolated by applying the Ribospin™ Plant Kit (GeneAll, Seoul, Republic of Korea), and it was subsequently transcribed into cDNA using the Tetro™ cDNA Synthesis Kit (Bioline Meridian Bioscience). The isolated DNA and cDNA were quantified using the NanoPhotometer^®^ P360 (Implen, Munich, Germany), and individual samples were adjusted to the same concentration.

The primers were designed based on the miRNA sequences using the database miRBase (https://mirbase.org/ (accessed on 14 September 2023)). For the family *Rosaceae*, the database does not contain the miRNA sequences for *P. pyraster*. Therefore, we selected the eight primer pairs already tested in our previous studies on different plant species (*Ginkgo biloba*, *Tilia cordata*, *Silybum marianum*, *Linum usitatissimum*). The following primers were used (Table 1).

The amplification result was checked on 3% agarose gels stained with GelRed^®^ nucleic acid in 10,000× in water (Biotium, San Francisco, CA, USA), running in 1 × TBE buffer at 100 V, 30 mA for 60 min. The ready-to-use GeneRuler Ultra Low Range DNA Ladder, Thermo Scientific™ (Waltham, MA, USA), was used. The samples with primers in which the polymorphism was recorded were separated on 15% PAGE gels (TBE-Urea Gels, Invitrogen, Waltham, MA, USA) and subsequently stained in a 1 × TBE solution containing GelRed^®^ nucleic acid for PAGE in 10,000× in water (Biotium). The gels were visualized on the G-Box Syngene electrophoresis documentation system and analyzed using GeneTools software version 4.3.10.0 (Syngene, Bengaluru, India).

### 2.8. PBA-Based Assay

PBA fingerprints were obtained following the basic protocol by Yamanaka et al. (2003) [43]. The PCR reactions were performed using the SureCycler 8800 (Agilent, Santa Clara, CA, USA). The MasterMix Robust HS Elizyme 2× (Elisabeth Pharmacon, Brno, Czech Republic) was used in the PCRs with 400 nmol/dm^−3^ of each primer. The following steps were carried out to obtain the thermal profile: 95 °C for 5 min, 45 cycles of 95 °C for 40 s, 50 °C for 45 s, 72 °C for 1 min, and a final elongation at 72 °C for 10 min. The PCR products were separated on 1.5% agarose gels stained with GelRed^®^ nucleic Acid Gel Stain (Biotium) and visualized by the BDA digital system 30 transilluminator (Analytik Jena, Jena, Germany).

### 2.9. PBA Expression Analysis

Actin ortholog locus of *Arabidopsis thaliana* (AT3G18780) was used as a housekeeping gene, based on the protocol by Liu et al. (2018) [44], to normalize the expression of the analyzed cytochrome P450 (NCBI—AF386512.1; Table 2) against the control variants of the leaves and roots to all analyzed stressed variants. qRT-PCR reactions were performed in MX3005P qPCR (Agilent). The reactions were performed in triplicate using the 2× Elizyme qPCR Master Mix (Elisabeth Pharmacon) with 10-fold diluted cDNA. The temperature and time conditions were as follows: 95 °C for 2 min, 40 cycles of 95 °C for 5 s, and 60 °C (actine) or 50 °C (P450) for 25 s with fluorescence reading.

PBA amplicons were separated in 3% AGE and scored for their length and presence and transformed into binary matrices. Qualitative analysis was performed by comparing the number of amplified amplicons, unique amplicons, and length of generated amplicons among the analyzed stressed variants in comparison to the control variant.

Cytochrome P450 expression quantification was performed according to Pfaffl et al. [45] and analyzed by the delta–delta Ct method. Standard curves for expression effectivity were prepared by 5 serial dilutions of the control-variant cDNA on 10-fold diluted cDNA. Melting curves of the generated amplicons were determined to check their specificity.

## 3. Results

### 3.1. Plant Growth and Mass Accumulation under Salt Treatment

**Figure 1:**
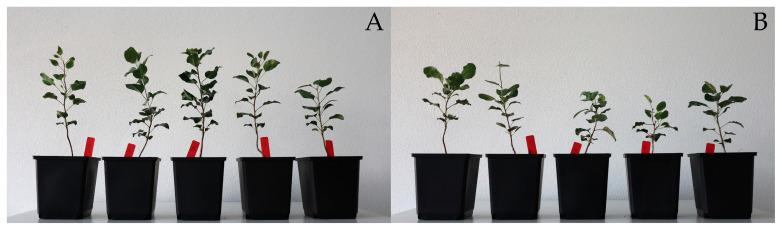
Samples of the control (**A**) and salt-treated (**B**) two-year-old seedlings of *P. pyraster* at the end of the experiment.

The salt treatment affected the growth and accumulation of the mass in the aboveground organs of the *P. pyraster* seedlings (Table 3, Figure 1). The stem increment was significantly reduced (−38%), as was the dry mass of the aboveground organs (DWS) (−37%). The growth and development of the leaves were negatively affected by the salt stress. The salt-treated seedlings formed smaller leaves, and the total leaf area (LA) (−31%) and dry matter of leaves (DWL) (−33%) were significantly reduced compared to the control plants. The experimental plants maintained a balanced specific leaf area (SLA) despite the salt treatment.

Within the experiment, the root growth was not significantly limited by the salinity of the substrate. The salt-treated seedlings maintained balanced values for most of the measured root parameters, including root length (RL), specific root length (SRL), root surface area (RSA), root volume (RV), average root diameter (ARD), number of root tips (NORT), and dry matter of root (DWR). The significant decrease in the root volume (−20%) was documented in the fraction of very fine roots (0–1 mm). Under the conditions of the substrate salinity, the seedlings invested mass to the root growth, which is documented by a significant increase in the root-to-shoot mass ratio (R:S = 0.9) compared to the control plants (R:S = 0.59).

**Figure 2:**
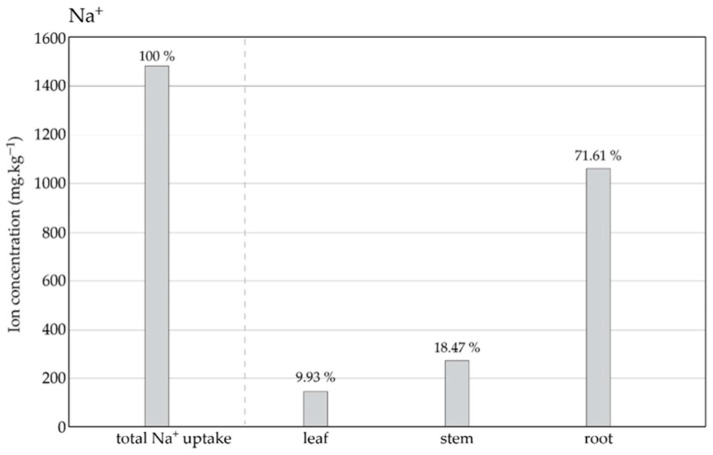
The content and distribution of Na+ ions in the plant tissues (leaves, stems, roots) of *P. pyraster* grown for 60 days under salt treatment with 100 mM NaCl solution. The values above the bars indicate distribution to the plant organs as a percentage of the total Na+ ion uptake.

The total ion concentration in the dry mass of the *P. pyraster* seedlings was 1500 mg·kg^−1^. Na^+^ ions were accumulated mainly in the roots (72%), and a significantly lower amount of Na^+^ was distributed to the aboveground organs, including stems (18%) and leaves (10%) (Figure 2). Under the salt treatment, the *P. pyraster* seedlings accumulated salt ions in the root, restricted their distribution to aboveground organs, and protected their leaves against intoxication and damage.

### 3.2. Leaf Water Status under Salt Treatment

**Figure 3:**
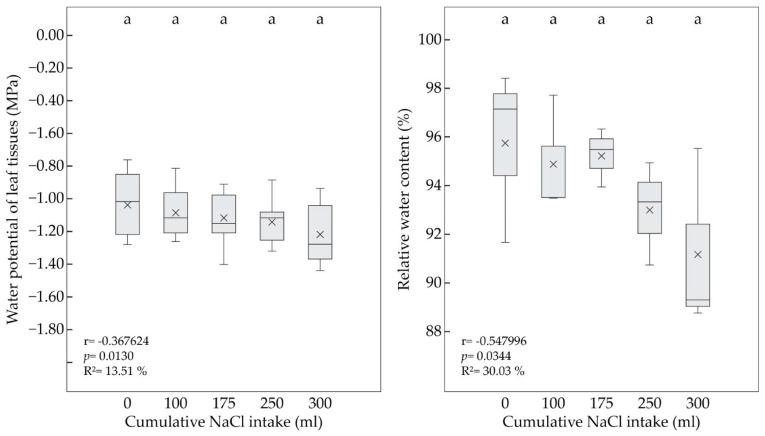
The water potential of leaf tissue (Ψ_wl_) (**left**) and relative water content (RWC) (**right**) of *P. pyraster* seedlings measured at the beginning of the experiment (0 mL) and during the salt treatment with 100 mM NaCl solution. A multiple comparison of the means (n = 9) was performed using the Tukey honest significant difference (HSD) test. The same letters indicate insignificant differences between measurements. The solid line indicates the median, the multiple symbol (×) shows the mean. The relationship between the plant parameters and the increasing volume of NaCl intake was analyzed by simple regression.

During the experiment, the two-year-old seedlings of *P. pyraster* maintained a balanced Ψ_wl_ (−1.12 ± 0.17 MPa), even at a higher level of the substrate salinity (300 mL per plant) (Figure 3, Left). The regression analysis confirmed a weak but significant relationship between increasing cumulative NaCl uptake and Ψ_wl_ (Figure 3, Left). Thus, a further drop of this parameter can be significant with the rising sum of NaCl intake. A significant, moderately strong relationship was also confirmed between the RWC values and the increasing volume of the cumulative NaCl intake. However, throughout the experiment, the RWC of the salt-treated plants ranged between 96 and 91%, without significant changes between the measurements (Figure 3, Right).

### 3.3. Effect of Salinity on Leaf Gas Exchange

**Figure 4:**
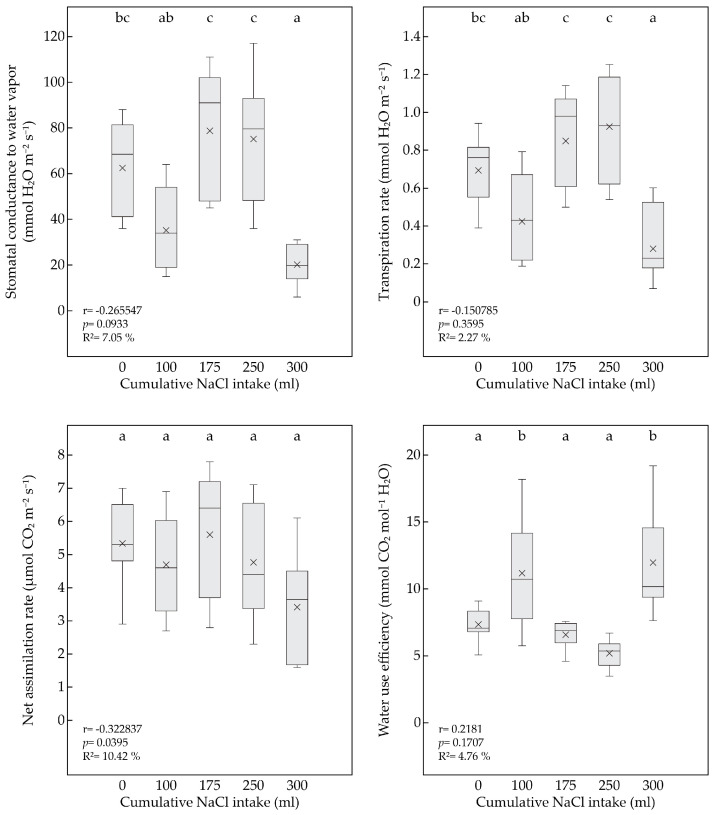
Box plot for the leaf gas exchange parameters (g_s_, E, A_n_, WUE) for seedlings of *P. pyraster* at the beginning of experiment (0 mL) and during the salt treatment with 100 mM NaCl solution. A multiple comparison of the means (n = 7) was performed using the Tukey honest significant difference (HSD) test. The different letters indicate significant differences between measurements. The solid line indicates the median, the multiple symbol (×) shows the mean. The relationship between the plant parameters and the increasing volume of NaCl intake was analyzed by simple regression.

Under the salt treatment, the *P. pyraster* seedlings maintained balanced values of the gas exchange parameters compared to the control plants for 50 days of the gradual increase in salt uptake into the substrate (Table 4). The seedlings increased their water use efficiency on day 20 of regular NaCl application and maintained a stable net photosynthetic rate (A_n_) per unit area (Figure 4). However, the gradual impact of salt stress on photosynthesis was documented by the negative correlation between the A_n_ values and the increasing volume of the cumulative NaCl intake (Figure 3). Significant decreases in the g_s_, E, and A_n_ parameters for the salt-treated plants were observed on day 60 of the experiment, when the cumulative NaCl intake was 300 mL per plant (Table 2).

### 3.4. Effect of Salinity on Plant Genome Response, Analyzed by DNA-Based Markers and Cytochrome P450 Expression 

**Figure 5:**
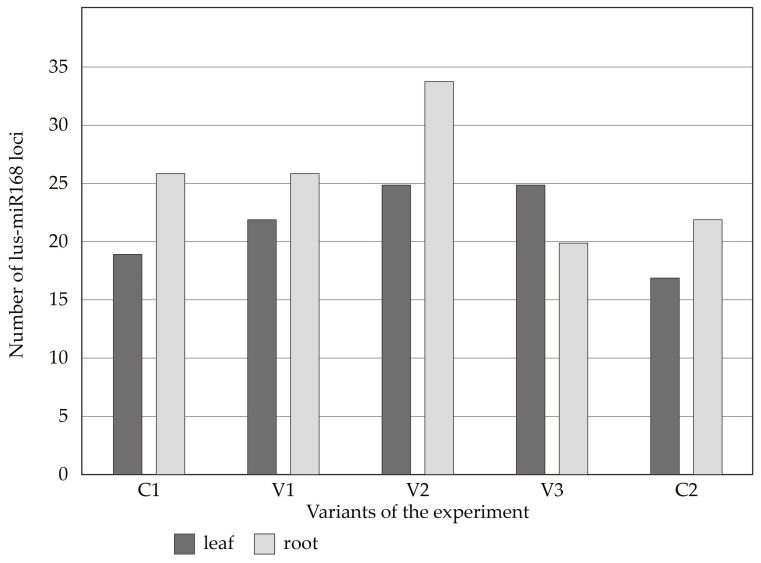
MiRNA loci detected by lus-miR168-based primers in leaf and root samples of the control (C1 and C2) and treatment (V1, V2 and V3) variants. C1—1st day of the experiment, without treatment; C2—60th day of the experiment, without treatment; V1—20th day of the experiment, 100 mL of 100 mM NaCl; V2—35th day of the experiment, 175 mL of 100 mM NaCl; V3—60th day of the experiment, 300 mL of 100mM NaCl.

**Figure 6:**
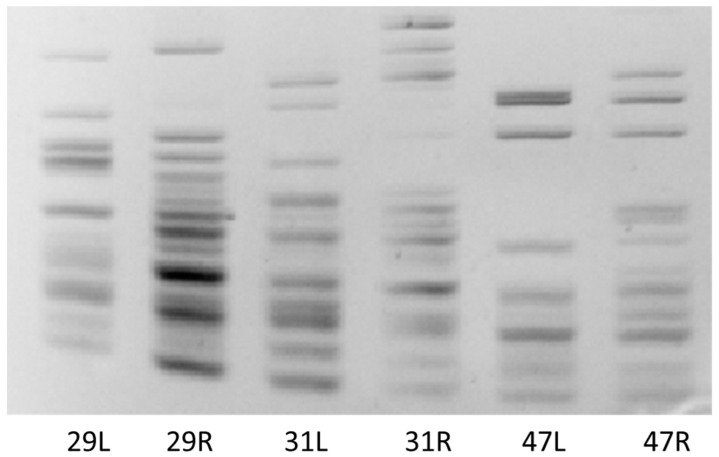
MiRNA-based stress-induced cDNA polymorphism detected by lus-miR168 primers. Representative figure of three biological replicates treated with 100 mM NaCl on 35th day of experiment. L—leaf sample, R—root sample. The numbers 29, 31, and 47 represent the three genotypes (biological replicates) of salt-treated seedlings.

Several miRNA-based primers were tested to detect the two-year-old *P. pyraster* seed-lings’ response to salt treatment. The primers lus-miR168, lus-miR408, gb-miR482, cca-miR396 and mdo-miR160 provided, to a certain extent, detection of the plant genome response to the applied stress conditions. From each of the control (C1 and C2) and treatment variants (V1, V2, and V3), three individual seedlings were analyzed. We observed individual miRNA loci profiles between the controls and the stress variants, but also within individual plants of the same variant. It seems that the individual seedlings representing three biological replicates reacted differently to the growth conditions. Because the experimental plants represent open-pollinated progenies, individual polymorphism was expected. By the lus-miR168-based primers, a total of 236 miRNA loci were detected, out of which 108 were detected in the leaf samples and 128 in the root samples (Figure 5 and Figure 6). As Figure 5 shows, the number of miR168-based loci in the root and leaf samples increased as the cumulative effect of the salt treatment grew, except in variant V3, where the number of miRNA loci increased only in the leaf samples. The presence of stress-sensitive miR168 loci in the control variants (C1 and C2) can reflect the genome response to the soil composition. The number of loci decreased in C2 in comparison to C1, possibly because these plants were watered throughout the whole experiment, which may have reduced the contents of some soil components.

The polymorphic miRNA-based loci provided by lus-miR168 among the individual seedlings were not only tissue-specific but also genotype-specific (Figure 6).

**Figure 7:**
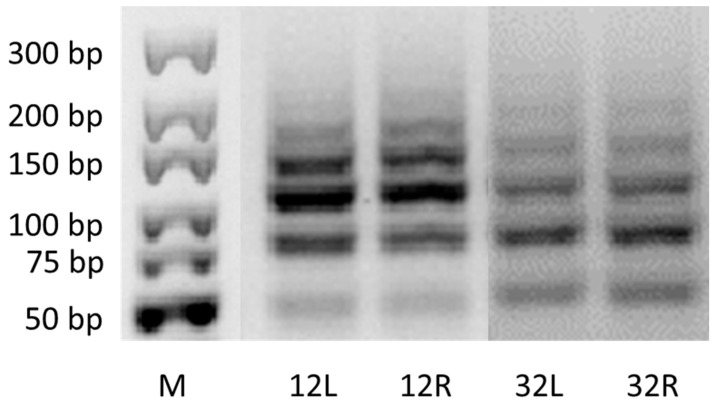
MiRNA loci detected by cca-miR160-based primers in both the analyzed tissue of control and treated variants. M—DNA size marker, L—leaf sample, R—root sample; 12 indicates the duplicated genotype of the control variant and 32 indicates the duplicated genotype of the treated seedlings.

The miR396 locus was amplified in all samples The profiles of the amplified mdo-miR160 loci were characterized by unique patterns (Figure 7). Regarding the control and treated samples, six loci, including one 50 bp, one < 75 bp, one < 100 bp, and three loci with a size of 100–150 bp were amplified.

Stress-sensitive lus-miR408 were reflected in the cumulative effect of salt treatment after 60 days of cultivation (300 mL of 100 mM NaCl), only in the leaf tissue in all three biological replicates. Lus-miR408 loci were amplified in the leaf and root tissues of some seedlings of the control samples and most seedlings of treated variants V1 (100 mL of 100 mM NaCl) and V2 (175 mL of 100 mM NaCl).

Analysis of the length polymorphism profiles was applied using the PBA technique, which maps cytochrome P450 sequences, which were used as a genomic marker system of the coding space.

The number of levels of amplified fragments obtained for the individual PBA primer combinations was in the range of 10–25 fragments, while only the CYP2BF+R profiles had a change in the plants growing under the salt stress compared to the control plants (Table 5).

In PBA expression, the obtained Cts in the analyzed leaf samples ranged from 29.21 to the 34.22 for actin, with standard deviations as follows: 20/100 mL 0.68; 35/175 mL 1.04; 60/300 mL 0.83. The obtained Cts in the analyzed root samples ranged from 34.04 to 37.35, with standard deviations as follows: 20/100 mL 0.49; 35/175 mL 1.04; 60/300 mL 0.74. The obtained Cts in the analyzed samples ranged from 29.2 to 30.54 in the leaf samples for P450 with standard deviations as follows: 20/100 mL 0.2; 35/175 mL 0.03; 60/300 mL 0.34, and from 29.65 to 30.39 in the root samples, with standard deviations as follows: 20/100 mL 0.2; 35/175 mL 0.31; 60/300 mL 0.06.

**Figure 8:**
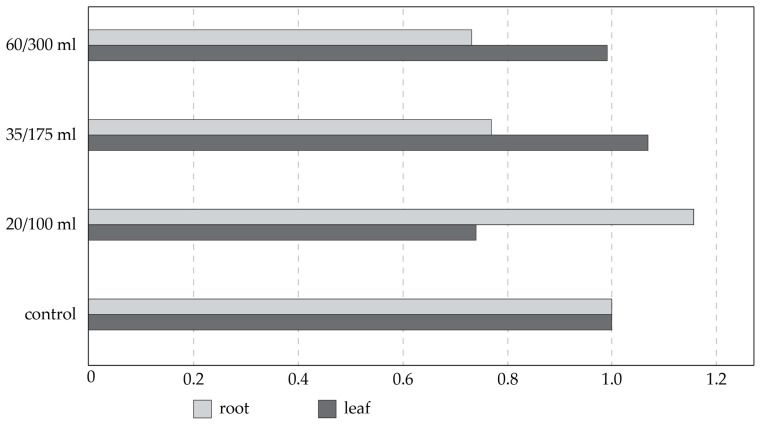
Comparison of expression ratios of *P. pyraster* cytochrome P450 in salt-stressed plants compared to control plants.

The activity of the cytochrome P450, due to the plant genome’s salt stress response, was found to be dose-dependent as well as tissue-type-dependent. However, none of these combinations in all three tested biological triplicates showed completely the same profiles (Figure 8). In the roots, the lower salt concentration showed expression ratios 0.43 times and 0.39 times higher, respectively, when compared to the higher salt concentration. In the leaves, the results were the opposite, and the higher salt concentrations showed expression ratios around 0.26 times higher on average when compared to the lower salt concentration.

**Figure 9:**
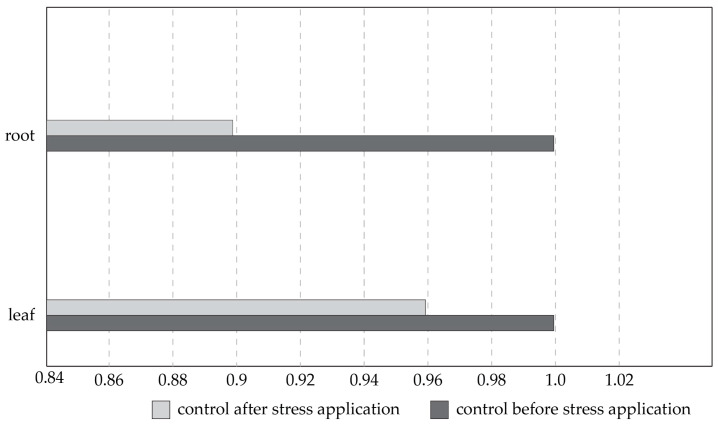
Comparison of expression ratios of *Pyrus pyraster* cytochrome P450 in control plants before and after the time of salt stress treatment.

A comparison of the *P. pyraster* cytochrome P450 expression levels of the control plants before and after the salt stress treatment was performed to obtain the data, and the expression of cytochrome P450 was stagnant or very similar over time without the stress factor of salt (Figure 9). For the roots, a 16% variance ranging between 0.9 and 1, and for the leaves, a 10% variance ranging between 0.96 and 1 were obtained, which shows stability in expression. 

## 4. Discussion

Salinity has a negative impact on plants, manifested in growth restriction, tissue damage, and physiological disorders [46,47,48]. Resistance to salinity stress and tolerance to different levels of salinity is species-specific. In woody plants, which are long-lived organisms, tolerance to salinity can change at different stages of their life cycle [49,50]. *Pyrus pyraster* is an adaptable woody plant growing in a wide range of habitats, so we focused on studying the reactions of *P. pyraster* seedlings to salt stress.

In our study, salinity affected the increment and accumulation of dry mass in the shoots of *P. pyraster* seedlings. The total leaf area (LA) and dry mass of the leaves were significantly reduced in the salt-treated plants. However, the root growth was not restricted, and the salt-treated seedlings maintained balanced values for most of the measured root parameters.

Seedlings invested mass to root growth, which is documented by the significant increase in the root-to-shoot mass ratio (R:S = 0.9). Investment in root growth and a higher spatial distribution of roots increase soil exploration and support water intake [20]. This is a typical response to salt stress associated with water stress [51]. The accumulation of mass in the roots was confirmed for *P. pyraster* under conditions of a regulated water regime, when seedlings were exposed to water scarcity [11,52]. Increasing the proportion of the root system mass in plants exposed to salinity has a positive effect through the increased retention of toxic salt ions in the root [51]. The obtained results show that *P. pyraster* seedlings blocked salt uptake in the root (72%) and stem (18%), and thus, protected the leaves from intoxication with sodium ions.

During the 60 days of salt treatment, the *P. pyraster* seedlings maintained a balanced Ψ_wl_, even at a higher level of the substrate salinity (300 mL per plant). The RWC of the salt-treated plants ranged between 96 and 91% without significant changes between measurements; however, a moderately strong negative relationship was confirmed between the RWC values and the increasing volume of the cumulative NaCl intake.

The ratio of the stomatal opening and the level of photosynthesis (water use efficiency, WUE) are used as indicators of plant tolerance to osmotic stress [51,53]. Under salt-stress situations, it is indicated that the leaves try to maintain high photosynthetic performance. The seedlings of *P. pyraster* responded to salinity by increasing their WUE. The increase in WUE was evident in two stages (on days 20 and 60) of the experiment, which is related to the increasing cumulative NaCl uptake per plant. After 20 days of salt treatment, the significant increase in the WUE was accompanied by a moderate, insignificant decrease in the g_s_ and E. After 60 days of salt treatment, a high concentration of salt ions in the substrate (300 mL per plant) induced a significant decrease in the g_s_ and E; however, the seedlings significantly increased their WUE and maintained a balanced net photosynthetic rate (A_n_).

Based on the obtained results and knowledge from previous research [12,13] we consider *P. pyraster* effective in its restriction of salt uptake. The salt-treated seedlings reduced their water loss in the initial (osmotic) phase of salt stress, increased their WUE, and maintained a steady rate of photosynthesis (A_n_) per unit area throughout the whole experiment.

These studies [12,13] performed in the early stages of growth show that *P. pyraster* can cope with increased salinity. To understand the effect of salt stress and the selection of salt-tolerant genotypes requires an interdisciplinary approach for the identification and characterization of markers at different levels (physiological, morphological, biochemical, and molecular) [54]. The stress triggers changes at the genomic, transcriptomic, and proteomic level, which can be detected by various types of sequence-specific primers. As many microRNAs affect trees’ adaptation to environmental conditions, they represent one of the key types of molecular markers also in connection with the identification of the functions of their target sequences [55]. Salt-responsive miRNAs in combination with simple sequence repeats (SSRs) were able to distinguish tolerant and susceptible rice genotypes [28].

MicroRNA molecules regulate the stress response of plants under different abiotic environmental factors [56,57]. Their regulation of plant salt-stress tolerance is intensively studied to support the development of miRNA-mediated salt-tolerant crops. MiRNAs that respond to salinity stress vary depending on the plant species, and their level of activity is species-specific [58]. The types of miRNAs tested in our study, miR160, miR168, miR408, and miR396, have been found to respond to salt stress in most plants [59]. MiR168 is considered to be a potential biomarker of abiotic stress [60]. One of the target sequences of the miR168 family are cytochrome P450 sequences, which are involved in a wide range of biosynthetic reactions [61]. The most polymorphic pattern of miR168-based marker reflects its sensitivity to detect the genome response to salt stress. The observation of the monomorphic miR396 amplification pattern is supported by the fact that miR396 regulates two transcription factors of the growth regulating factor (GRF) class and GRF-interacting factor (GIF) transcriptional co-regulators [62,63], which control the growth and development of plants. So, its activity is essential for plants to adjust to their environmental conditions. However, miR396 is upregulated by various stress conditions, such as drought and UV-B irradiation [64,65], and its overexpression inhibits growth but enhances survival in plants subjected to salt and cold stress [66,67]. Conserved types of miRNA families, where miR160 belongs, play a significant role in the regulation of plant growth processes. This observation can be explained by the fact that the miR160 family targets the auxin response factor (ARF) gene involved in the auxin pathway, which is essential for the growth and development of plants [68]. Increased miR408 activity corresponds to increased tolerance to salinity, cold, and oxidative stress, and to increased sensitivity to drought and osmotic stress [69]. In our observations, the cumulative effect of salinity stress was detected by lus-miR408 mainly in the leaves. Tissue-specific miRNA activity from salt stress has been observed in different tree species [70,71].

We can conclude that each seedling reacted to stress conditions individually, where we observed a genotype-specific reaction of the genome to stress conditions. This applies to both the control and stress variants (Figure 6). We also observed the reaction of stress-sensitive miRNAs to growing conditions in the control samples, although to a lesser extent than in the seedlings watered with saline solution. This means that the composition of the growing substrate caused the reaction of stress-sensitive markers. A more sensitive reaction was observed in the root part (this applies to V1 and V2); in V3, there was a cumulation of the reaction to stress, especially in the leaf part. This is true for most markers used (Figure 5).

Other types of genes and molecules are involved in the stress response of plants. The P450 gene superfamily is the largest family of plant enzyme proteins, and they have diverse functions in various biochemical reactions, including the biosynthesis of flavonoids, abscisic acid, phenylpropanol, and brassinolide. Most of the P450 genes in the same family or subfamily have similar functions, whereas the genes from different families show distinct functional roles [72]. Here, molecular markers based on P450-Based Analog (PBA), a type of functional genomic markers [43], were used to analyze the fingerprints of control and salinity-stressed plants pf *Pyrus pyraster*. Only one of three tested primer combinations showed polymorphic profiles, and this was in the case of stressed roots of *P. pyraster*, where insertions in the obtained PBA fingerprint pattern were detected. Thus, comparing these types of markers with the miRNA markers applied in this study, the PBA marker technique was not able to analyze changes under the stress accurately.

qRT-PCR is a very sensitive and fully reproducible molecular technique. It provides a wide range of quantification strategies that allow the analysis of individual parts of multiplex gene expression [73,74,75,76,77]. A first step in precise and reproducible gene expression quantification is normalization to the expression levels of the verified target reference gene/s that should be proven to have only minor differences in their expressions in various developmental stages, tissue or organ types, or experimental conditions [77,78]. Many different reference genes have been tested to be suitable for qRT-PCR analysis in pears, such as GAPDH, EF1a, TUB, ACT, GAPC, SKD1, UBQ5, and YLS8 [44,79,80,81]. Some of them were previously reported to have highly stable expression levels in pear in different experimental conditions [18,82,83]. Actin, which was chosen for our study, was widely used previously in qRT-PCR gene expression analysis in plants [83,84], and in our analysis, its expression stability was confirmed in the control plants before and after salt stress treatment sampling.

The qRT-PCR technique was chosen to quantify gene expression profile changes in response to developmental transitions (fruit development, anthocyanin biosynthesis, or sclereid formation,) and environmental changes (dehydration stress, low temperature, or biotic stress) in pear [80,85,86,87,88,89]. Here, the expression of cytochrome P450 due to the plant genome’s salt stress response was found to be a dose-dependent as well as tissue-type-dependent, corresponding to the phase of osmotic stress at the beginning and then to the phase of coping with stress. Previously, qRT-PCR was conducted to analyze the P450 gene expression patterns of cotton under salt stress, and a total of 55 differentially expressed P450 family genes were identified [90], which corresponds with our results. The *AtCYP709B3* gene was found to be highly expressed under salt stress in *Arabidopsis*, providing tolerance to salinity [91]. An increased expression of the *CYP709* family was also found in salt-stressed *Robinia pseudoacacia* [92]. 

## 5. Conclusions

The results of the present study document the ability of *P. pyraster* seedlings to withstand salinity stress. Young plants invested in root growth, increased the root-to-shoot ratio, and retained a substantial portion of salt ions in the root. The seedlings were effective in controlling the movement of Na^+^ ions into the leaves, thus protecting them from intoxication and damage. Under conditions of increasing salinity, *P. pyraster* seedlings maintained a balanced water regime of the leaf tissues. In response to osmotic stress, they reduced the leaf area, formed smaller leaves, effectively regulated the stomatal conductance (g_s_), and increased the water use efficiency (WUE). During the experiment, they maintained a stable net rate of photosynthesis (A_n_) per unit area. A decrease in the leaf gas exchange parameters (g_s_, E, A_n_) was manifested at the end of the experiment compared to the control, when the cumulative NaCl intake was 300 mL per plant.

From two types of methods based on the cytochrome P450 genes, only qRT-PCR was applicable for salt-stress-conditioned changes in the roots and leaves of *P. pyraster*, where in the roots, a lower salt concentration showed higher expression ratios, and in the leaves, the results were the opposite, and higher salt concentrations showed higher expression ratios. The expression of the P450 family under abiotic stresses needs further study, as the identification and analysis of the P450 gene family will be helpful for breeding salt-tolerant plant varieties. MicroRNA-based markers reflected a sensitive genome response to salt-stress conditions. In lus-miR168, tissue-specific and genotype-specific reactions were detected. The cumulative effect of the salt treatment was observed mostly in the leaf tissues, which corresponds to some physiological markers analyzed in the experiment. Our observations confirm that miR160, miR168, miR408, and miR396 respond to salt stress in pear. 

## Figures and Tables

**Table 1:** Sequences of primers used (5′-3′).

Primer	Sequences (5′-3′)
lus-miR168_F	CAC GCA TCG CTT GGT GCA GGT
lus-miR168_R	CCA GTG CAG GGT CCG AGG TA
lus-miR408a_F	GGC TGG GAA CAG ACA GAG CAT GGA
lus-miR408a_R	GGG AAA AAG GCC AGG GAA GAG G
cca-miR156a_F	TGA CAG AAG AGA GTG AGC AC
cca-miR156a_R cca-miR396a_F	GTG CTC ACT CTC TTC TGT CA TTC CAC AGC TTT CTT GAA CTT
cca-miR396a_R gb-miR482_F	GTT CAA GAA AGC TGT GGG AAA TGG GTT GTA GTC TTC AGG AGT GGG
gb-miR482_R	GAA GGC AAT AGG AAT GGG AGG ATC
mdo-miR398_F	TGT GTT CTC AGG TCA GGG GTT
mdo-miR398_R	AAC CCC TGA CCT GAG AAC ACA
mdo-miR160a_F	TGC CTG GCT CCC TGT ATG CCA
mdo_miR160a_R	TGG CAT ACA GGG AGC CAG GCA
mdo-miR172a_F	AGA ATC TTG ATG ATG CTG CAT
mdo-miR172a_R	ATG CAG CAT CAT CAA GAT TCT

Notes: lus—*Linum usitatissimum*, cca—*Cynara cardunculus*, gb—*Ginkgo biloba*, mdo—*Malus domestica*.

**Table 2:** Primers of gene of interest and housekeeping gene primers used for the analysis.

Primer Name	Primer Sequence
PC Act F1/R1	F:CTCCCAGGGCTGTGTTTCCTA
	R:CTCCATGTCATCCCAGTTGCT
PC P450 F1/R1	F:GAACTCTTGAGGCACCCGAA
	R:AATGGGGCAACTGGGTGTAG

**Table 3:** The growth parameters and biomass allocation of the two-year-old seedlings of *P. pyraster* in the pot experiment after 60 days of salt treatment. A multiple comparison of the means (n = 12) was performed using the 95% Tukey honest significant difference (HSD) test. Data are the mean values and standard deviations (±SD). Mean values followed by different letters are significantly different (*p* < 0.05).

Parameter	*p*-Value	Control	100 mM
Stem length (mm)	0.0024	232.75 (±58.67) a	160.33 (±43.83) b
Stem increment (mm)	0.0023	139.25 (±46.04) a	85.83 (±27.75) b
LA (mm^2^)	0.0245	11,876.10 (±4301.85) a	8216.16 (±3011.44) b
SLA (mm^2^·mg^−1^)	0.7016	16.57 (±1.61) a	16.80 (±1.40) a
DW (mg)	0.0037	1426.75 (±483.07) a	893.50 (±302.38) b
DWL (mg)	0.0114	717.92 (±241.29) a	484.50 (±166.32) b
DWS (mg)	0.0023	708.83 (±258.55) a	409.00 (±154.36) b
LWC (%)	0.1228	60.47 (±1.92) a	61.59 (±1.33) a
RL (mm)	0.1374	7312.79 (±1999.09) a	6321.61 (±752.54) a
SRL (mm·mg^−1^)	0.1705	9.54 (±2.10) a	8.37 (±1.94) a
RSA (mm^2^)	0.1707	7459.93 (±1191.91) a	6711.47 (±1213.18) a
RV (mm^3^)	0.1686	2424.01 (±726.21) a	2054.15 (±480.23) a
ARD (mm)	0.8199	0.42 (±0.05) a	0.41 (±0.04) a
NORT	0.7285	1793.00 (±290.89) a	1852.83 (±490.04) a
DWR (mg)	0.5940	740.27 (±169.35) a	705.46 (±129.51) a
R:S	0.0057	0.59 (±0.20) a	0.90 (±0.28) b
Fine root volume (mm^3^)	0.1044	853.83 (±168.90) a	720.92 (±203.09) a
Volume of coarse roots (mm^3^)	0.4846	1394.99 (±347.51) a	1272.78 (±436.85) a
Volume of very fine roots 0–1 (mm^3^)	0.0350	575.95 (±152.73) a	460.03 (±92.82) b
Fine-to-coarse root ratio	0.4532	0.62 (±0.11) a	0.56 (±0.25) a

LA—leaf area; SLA—specific leaf area; DW—dry mass of shoots; DWL—dry mass of leaves; DWS—dry mass of the stem; LWC—leaf water content; RL—root length; SRL—specific root length; RSA—root surface area; RV—root volume; ARD—average root diameter; NORT—number of root tips; DWR—dry mass of roots; R:S—root-to-shoot mass ratio.

**Table 4:** Leaf gas exchange parameters measured in the leaves of *P. pyraster* seedlings during 60 days of salt treatment and comparison with control plants (n = 7). Mean values followed by different letters are significantly different (*p* < 0.05).

CSI (mL.Plant^−1^)	Parameter	*p*-Value	g_s_ (mmol H_2_O m^−2^ s^−1^)	*p*-Value	A_n_ (μmol CO_2_ m^−2^ s^−1^)	*p*-Value	E (mmol H_2_O m^−2^ s^−1^)	*p*-Value	WUE (mmol CO_2_ mol^−1^ H_2_O)
0	Control	0.9736	63.00 (±37.36) a	0.7003	5.00 (±2.24) a	0.5976	0.63 (±0.31) a	0.1277	9.36 (±3.91) a
	100 mM		62.50 (±20.91) a		5.34 (±1.42) a		0.69 (±0.19) a		7.34 (±1.17) a
100	Control	0.1389	57.43 (±31.93) a	0.2885	5.55 (±1.54) a	0.3598	0.57 (±0.30) a	0.8752	10.77 (±5.26) a
	100 mM		35.29 (±18.62) a		4.69 (±1.50) a		0.42 (±0.24) a		11.16 (±4.27) a
175	Control	0.0662	110.57 (±29.90) a	0.1467	7.01 (±1.37) a	0.0712	1.11 (±0.24) a	0.9962	6.57 (±2.06) a
	100 mM		78.71 (±29.07) a		5.60 (±1.99) a		0.85 (±0.26) a		6.57 (±1.06) a
250	Control	0.0758	105.14 (±32.92) a	0.2742	5.79 (±1.71) a	0.0676	1.21 (±0.30) a	0.6642	4.90 (±1.37) a
	100 mM		75.38 (±26.86) a		4.76 (±1.75) a		0.92 (±0.27) a		5.17 (±1.01) a
300	Control	0.0143	44.5 (±12.82) a	0.0166	4.94 (±1.26) a	0.0178	0.56 (±0.23) a	0.2482	9.66 (±3.10) a
	100 mM		24.13 (±13.43) b		3.02 (±1.31) b		0.28 (±0.16) b		11.95 (±3.91) a

CSI—cumulative NaCl intake per plant; A_n_—net assimilation rate; E—transpiration rate; g_s_—stomatal conductance to water vapor; WUE—water use efficiency.

**Table 5:** Characteristics of PBA amplification profiles of analyzed pear variants.

		Stress/Primer Combination	CYPA1F+R *	CYP2BF+R	CYP2CF+R
Length range of amplified fragments	leaves	control before	30–900 bp	40–1000 bp	80–1000 bp
		20/100 mL			
		35/175 mL			
		60/300 mL			
		control after			
	roots	control before	180–700 bp	90–1000 bp	50–1200 bp
		20/100 mL		170–1000 bp	
		35/175 mL		210–1000 bp	
		60/300 mL		170–1000 bp	
		control after		90–1000 bp	
Type of obtained profile	leaves	control before	all monomophic		
		20/100 mL			
		35/175 mL			
		60/300 mL			
		control after			
	roots	control before	monomorphic		
		20/100 mL	monomorphic	polymorphic	monomorphic
		35/175 mL	monomorphic	polymorphic	monomorphic
		60/300 mL	monomorphic	polymorphic	monomorphic
		control after	monomorphic		
Unique fragments	leaves	control before	no		
		20/100 mL			
		35/175 mL			
		60/300 mL			
		control after			
	roots	control before	no	no	no
		20/100 mL	no	yes/220, 790 bp insertion	no
		35/175 mL	no	yes/220 bp insertion	no
		60/300 mL	no	yes/670 bp insertion	no
		control after	no	no	no
Difference in PBA profile of leaves/roots	-	-	no	yes	no
Difference in PBA profile of control/stress variants	-	-	no	yes	no

* Primer abbreviations are as reported in reference [43].

## Data Availability

Data are contained within the article.
